# L-Glutamine Supplementation Improves the In Vitro Qualitative Parameters of Cryopreserved Qinchuan Bull Sperm

**DOI:** 10.3390/ani15203052

**Published:** 2025-10-21

**Authors:** Benshun Yang, Li Liu, Nanfei Wang, Zhenghai Zhou, Zhipeng Zhang, Yuan Li, Linsen Zan, Wucai Yang

**Affiliations:** 1 College of Animal Science and Technology, Northwest A&F University, Yangling 712100, Chinazzh18755044693@163.com (Z.Z.); 19860911362@163.com (Z.Z.);; 2 College of Animal Science and Technology, Huazhong Agricultural University, Wuhan 430070, China; 3 Shenzhen Research Institute, Northwest A&F University, Shenzhen 518000, China

**Keywords:** L-glutamine, semen cryopreservation, cattle, oxidative damage, metabolomics

## Abstract

**Simple Summary:**

For ease of storage and transportation, semen is typically preserved by freezing. However, cryopreservation inevitably exposes sperm to oxidative stress and other damages, leading to reduced sperm quality. Thus, exploring the mechanisms underlying sperm freezability and developing novel sperm cryoprotectants are urgent priorities. In this study, metabolomic analysis was used to characterize metabolite changes in semen with high and low freezability. The results revealed significant differences in metabolite composition between semen with high freezability and that with low freezability, with differential metabolites primarily enriched in amino acid metabolism and lipid metabolism pathways. Notably, among these differential metabolites, the abundance of L-glutamine (L-Gln) in the high freezability group was significantly higher than that in the low freezability group. Based on this, we further investigated the role of L-Gln in sperm freezability and found that supplementation with L-Gln significantly improved freeze–thaw sperm motility, acrosome and plasma membrane integrity, as well as enhanced antioxidant capacity and energy metabolism. This suggests that L-Gln holds promise as an endogenous cryoprotectant to alleviate sperm damage during cryopreservation. These findings provide a theoretical basis for improving semen freezability and contribute to enhancing reproductive efficiency in livestock production.

**Abstract:**

Semen cryopreservation is a crucial technology for enhancing reproductive efficiency in livestock production; however, oxidative stress-induced sperm damage during the freeze–thaw process remains a significant challenge. In this study, metabolomics was used to analyze the differences in metabolites in semen from Qinchuan cattle with different freezing tolerance, and to screen out the candidate markers of sperm freezing tolerance. The metabolomics results indicate that a total of 264 differential metabolites were identified, and KEGG pathway annotation revealed that amino acid metabolism (15.07%) were prominently represented, and L-glutamine (L-Gln) showed a particularly high abundance in high freezability group (HFG) compared to the low freezability group (LFG). Further experiments demonstrated that L-glutamine supplementation significantly improved post-thaw sperm motility, plasma membrane integrity, and acrosomal integrity (*p* < 0.05). It also enhanced sperm antioxidant capacity by increasing the activities of superoxide dismutase (SOD), glutathione peroxidase (GSH-Px), and catalase (CAT), while reducing malondialdehyde (MDA) content (*p* < 0.05). Additionally, L-Gln maintained mitochondrial function and energy homeostasis by elevating mitochondrial membrane potential (MMP) and promoting AMPK phosphorylation (*p* < 0.05). These results indicate that L-glutamine alleviates oxidative damage during cryopreservation and enhances semen freeze tolerance.

## 1. Introduction

Semen cryopreservation is a critical reproductive management technology in livestock production. When combined with artificial insemination, this technique can overcome the spatiotemporal constraints of livestock breeding and mitigate cross-infection risks, thereby significantly improving reproductive efficiency [1]. However, cryopreservation inevitably damages sperm [2,3]. Oxidative stress is one of the most critical contributors to cryodamage [4]. Under oxidative stress, excessive production and accumulation of reactive oxygen species (ROS) within sperm cells trigger a cascade of adverse effects, including lipid peroxidation, compromised acrosomal and plasma membrane integrity, mitochondrial dysfunction, DNA damage and sperm apoptosis [5]. In addition, the formation of intracellular ice crystals and fluctuations of osmotic pressure during cryopreservation are likely to induce mechanical damage to biological membranes, subsequently impairing sperm motility and fertilization capacity [6]. These negative effects severely hinder the widespread adoption of semen cryopreservation technology. Therefore, it is essential to investigate the mechanism of sperm cryotolerance and develop effective cryoprotectants to reduce oxidative damage.

Recent studies have explored the effects of adding adjuvants such as plant extracts, trace elements, and antioxidants to semen cryopreservation solutions on sperm motility. For example, grape seed extracts such as proanthocyanidins, resveratrol and naringin as well as the trace element boron can effectively reduce cryopreservation-induced oxidative stress and maintain mitochondrial membrane potential [7,8,9,10]. Despite their positive effects, these additives pose challenges in terms of biocompatibility and stability [11,12], and the specific mechanisms by which they alleviate cryodamage remain to be further elucidated.

With the advancement of multi-omics technologies, metabolomics has been applied to investigate metabolic changes in sperm during cryopreservation, providing crucial insights into the underlying mechanisms of sperm cryotolerance [13,14]. For instance, metabolomic profiling of Holstein bull semen revealed significant changes in seminal plasma and sperm metabolites before and after cryopreservation [15]. Another metabolomic study on Holstein bull seminal plasma demonstrated that fructose is the most abundant metabolite among all seminal plasma components, with its concentration significantly higher in bulls with high fertility than those with low fertility [16]. Although these studies have provided valuable information on the metabolome of bull semen, research on the metabolome of beef bull semen remains limited. In this study, we selected Qinchuan cattle, a well-known native beef cattle breed in China, as the experimental subjects. This breed not only exhibits good growth performance and high-quality meat, but also has strong adaptability to various environmental conditions, thus playing an important role in both beef cattle scientific research and commercial beef production. However, at present, the factors related to semen cryopreservation tolerance in native beef cattle such as Qinchuan cattle have not been fully explored. L-Glutamine (L-Gln) is a non-essential amino acid widely present in humans and animals. By promoting glutathione (GSH) synthesis and activating the Nrf2 signaling pathway, L-Gln enhances cellular antioxidant capacity and mitigates ROS-induced oxidative damage [17,18]. Additionally, L-Gln participates in amino acid metabolism and the tricarboxylic acid (TCA) cycle, providing energy for mitochondria [19]. These critical functions suggest L-Gln’s potential involvement in sperm cryotolerance. Building on this foundation, our study identified L-Gln as a significantly enriched metabolite in cryotolerant semen of Qinchuan cattle. Further experimental validation explored the role of L-Gln as an endogenous cryoprotectant in enhancing sperm antioxidant capacity, plasma membrane integrity, acrosome structure, and mitochondrial function, offering a potent strategy to mitigate oxidative stress during sperm cryopreservation.

## 2. Materials and Methods

All experimental protocols in this study were approved by the Laboratory Animal Management Committee of Northwest A&F University (Yangling, Shaanxi, China).

### 2.1. Semen Collection and Quality Assessment

Semen samples were collected from eight healthy Qinchuan bulls, aged 3 to 5 years, with excellent reproductive performance, housed at the National Beef Cattle Improvement Center of Northwest A&F University. The semen collection was concentrated on spring and autumn to avoid the adverse effects of summer heat stress on sperm quality. During this period, the average ambient temperature was 16~18 °C, and the average relative humidity was 55~65%. Semen was collected twice per week using an artificial vagina, with a semen volume of 6.5 ± 0.8 mL per collection for each bull. All bulls were maintained under consistent feeding and management conditions.

Semen quality was assessed using a fully automated sperm quality analyzer (SQA-Vb, Caesarea, Israel). The parameters assessed included sperm motility (%), progressive motility (%), motile sperm concentration (million/mL), progressively motile sperm concentration (million/mL), velocity (μm/s) and sperm abnormality rate (%).

### 2.2. Semen Freezing and Grouping

Semen samples meeting the criteria of sperm motility ≥ 70%, sperm concentration ≥ 6 × 10^8^/mL, and sperm abnormality rate ≤ 15% were selected for freezing. Fresh semen was diluted using a one-step dilution method by mixing with preheated (37 °C) semen diluent (IMV, L’Aigle, France) to achieve a final sperm concentration of 5 × 10^7^/mL. The diluted semen was equilibrated at 4 °C for 4 h and packaged into straws using an automatic straw filling and sealing machine (IMV, France). Semen freezing was performed using the liquid nitrogen vapor freezing method. Straws were suspended 5 cm above the liquid nitrogen surface for 15 min, followed by immersion in liquid nitrogen for storage. After 7 days of storage in liquid nitrogen, semen straws were thawed in a 37 °C water bath for 30 s. Post-thaw sperm quality was assessed using the sperm quality analyzer. Relative sperm progressive motility was calculated using the following formula, and semen samples were categorized into the high freezability group (HFG) and the low freezability group (LFG). Relative sperm progressive motility = (frozen-thawed sperm progressive motility)/(fresh sperm progressive motility) × 100%.

### 2.3. Metabolomic Analysis

In this study, semen samples were collected from 4 Qinchuan bulls in each of the HFG and the LFG, with each bull providing 2–3 ejaculate samples and each ejaculate sample being processed and analyzed independently. Specifically, the first bull provided 2 samples, the second bull 2 samples, the third bull 3 samples, and the fourth bull 3 samples, resulting in a total of 10 samples per group for metabolomic analysis. Metabolite analysis was performed using liquid chromatography-mass spectrometry (LC-MS).

For each sample, 100 μL of semen was mixed with 400 μL of 80% methanol-water solution, vortexed, and incubated in an ice bath for 5 min. The mixture was then centrifuged at 15,000× *g* for 20 min at 4 °C. A portion of the supernatant was diluted with LC-MS-grade water to adjust the methanol concentration to 53%. After a second centrifugation at 15,000× *g* for 20 min at 4 °C, the supernatant was collected and analyzed using LC-MS.

Chromatography conditions: A Hypersil Gold C18 column was used with a column temperature set at 40 °C and a flow rate of 0.2 mL/min. Mobile phase A was 0.1% formic acid in water, and mobile phase B was methanol. Mass spectrometry conditions: The scan range was m/z 100–1500. The spray voltage was set at 3.5 kV, sheath gas flow rate at 35 psi, auxiliary gas flow rate at 10 L/min, ion transfer capillary temperature at 320 °C, S-lens RF level at 60, and auxiliary gas heater temperature at 350 °C. Both positive and negative ion modes were used. MS/MS analysis was performed using data-dependent scans.

Metabolites were annotated using the Kyoto Encyclopedia of Genes and Genomes (KEGG, https://www.genome.jp/kegg/pathway.html, accessed on 15 January 2024) and the Human Metabolome Database(HMDB, https://hmdb.ca/metabolites, accessed on 15 January 2024). Multivariate statistical analysis: Metabolomics data were processed using the software metaX v2.0.0. Principal Component Analysis (PCA) and Orthogonal Projections to Latent Structures-Discriminant Analysis (OPLS-DA) were employed to analyze the data structure characteristics of samples and differences between groups. Variable Importance in Projection (VIP) values were calculated based on the OPLS-DA model for the subsequent screening of differential metabolites. Univariate statistical analysis: A t-test was used to calculate the statistical significance (*p*-value) of metabolites between the two groups. Fold change (FC) values were computed to represent the relative differences between groups. Visualization: Volcano plots were created using the R package ggplot2 v3.4.3, integrating VIP values, log_2_(fold change), and −log_10_(*p*-value) to identify key metabolites. Heatmaps were generated using the R package Pheatmap, with metabolite data normalized by z-scores.

### 2.4. Detection of Sperm Membrane Integrity

A stock solution of L-Gln (25 mg/mL) was prepared by dissolving 100 mg of L-Gln (Solarbio, China) in 4 mL of ultrapure water, and added to the diluted semen to achieve final concentrations of 0, 0.3, 0.6, 0.9, and 1.2 mg/mL. Specifically, for each 1 mL of diluted semen, 0, 12, 24, 36, or 48 μL of the stock solution was added. All additions were performed immediately prior to equilibration at 4 °C.

Sperm plasma membrane integrity was evaluated using the Hypo-osmotic Swelling Test (HOST) as described by Correa [20]. Briefly, 0.6756 g of fructose and 0.3676 g of glucose (both from Solarbio, Beijing, China) were dissolved in double-distilled water and diluted to 50 mL, with the osmolarity adjusted to 150 mOsm/L.

A 100 µL of frozen-thawed semen was mixed with 1 mL of the hypo-osmotic solution and incubated at room temperature for 30 min. A 10 µL aliquot was then examined under a microscope, with at least 100 sperm cells counted per field. Sperm with bent tails were considered to have intact membranes. The sperm tail bending rate was calculated as follows: sperm tail bending rate = (bent-tail sperm count)/(total sperm count) × 100%.

### 2.5. Detection of Sperm Acrosome Integrity

Acrosome integrity was evaluated using Fluorescein Isothiocyanate-labeled Peanut Agglutinin (FITC-PNA) staining. A 50 µL sample of thawed semen was spread on a glass slide, air-dried, and fixed with anhydrous methanol for 10 min. After drying, the slide was incubated with 30 µL of FITC-PNA staining solution (Sigma, Darmstadt, Germany) at 37 °C in the dark for 30 min. After 2–3 washes with PBS, the slide was air-dried and mounted with a sealing medium (Solarbio, China). Under a fluorescence microscope, at least 100 sperm cells per field were counted. Sperm exhibiting uniform green fluorescence on the acrosomal region were considered intact. The Acrosome Integrity Rate was calculated as follows: acrosome integrity rate = (intact acrosome sperm count)/(total sperm count) × 100%.

### 2.6. Detection of Semen Antioxidant Capacity

The activities of CAT, GSH-Px, and SOD in frozen semen were measured using commercial assay kits (Nanjing Jiancheng Biological Engineering Co., Ltd., Nanjing, China), following the manufacturer’s instructions. Additionally, the MDA content was determined using an MDA assay kit, and total antioxidant capacity (T-AOC) was assessed using a T-AOC kit (Solarbio, China).

### 2.7. Detection of ATP Content and Mitochondrial Membrane Potential in Semen

ATP content and mitochondrial membrane potential (MMP) in frozen semen were measured using ATP and MMP assay kits, respectively (Solarbio, China). All procedures were performed according to the manufacturers’ protocols.

### 2.8. Western Blot Analysis

Sperm proteins were extracted using RIPA lysis buffer (Solarbio, China) and quantified with a BCA assay kit (Solarbio, China). Equal protein amounts (normalized to the lowest concentration) were mixed with loading buffer (Solarbio, China) and denatured by boiling at 100 °C for 10 min. Proteins were separated by SDS-PAGE at 120 V for 1 h and transferred to a PVDF membrane. The membrane was washed with TBST (three times for 5 min each) and blocked with 5% blocking solution (Solarbio, China) for 30 min. It was then incubated overnight at 4 °C with primary antibodies against phosphorylated AMPK (P-AMPK, 1:1000, Beyotime, Shanghai, China), AMPK (1:1000, Beyotime, China), and β-actin (1:2000, Abcam, UK) diluted in antibody dilution buffer (Solarbio, China). After three washes with TBST, the membrane was incubated with HRP-conjugated secondary antibodies (Solarbio, China) at room temperature for 2 h in the dark, followed by three washes with TBST. Bands were visualized using a chemiluminescent substrate (Millipore, Burlington, MA, USA) and the Gel Doc XR+ system (Bio-Rad, Hercules, CA, USA).

### 2.9. Statistical Analysis

Data were tested for normality and homogeneity of variances. One-Way ANOVA was conducted using SPSS v25.0, and if significant, Tukey’s HSD test was used for post hoc comparisons. Results are expressed as mean ± SD, with significance set at *p* < 0.05. Different lowercase letters indicate significant differences (*p* < 0.05), while the same lowercase letters indicate no significant differences (*p* > 0.05).

## 3. Results

### 3.1. Classification of Semen into HFG and LFG

Fresh semen of Qinchuan bulls with similar motility was selected for freezing. Based on the relative sperm progressive motility, semen was categorized into a high freezability group (HFG) and a low freezability group (LFG). Sperm quality assessments showed that both sperm motility and progressive motility were significantly higher in the HFG compared to the LFG (*p* < 0.05; Table 1).

### 3.2. Classification of Metabolites Between HFG and LFG

**Figure 1:**
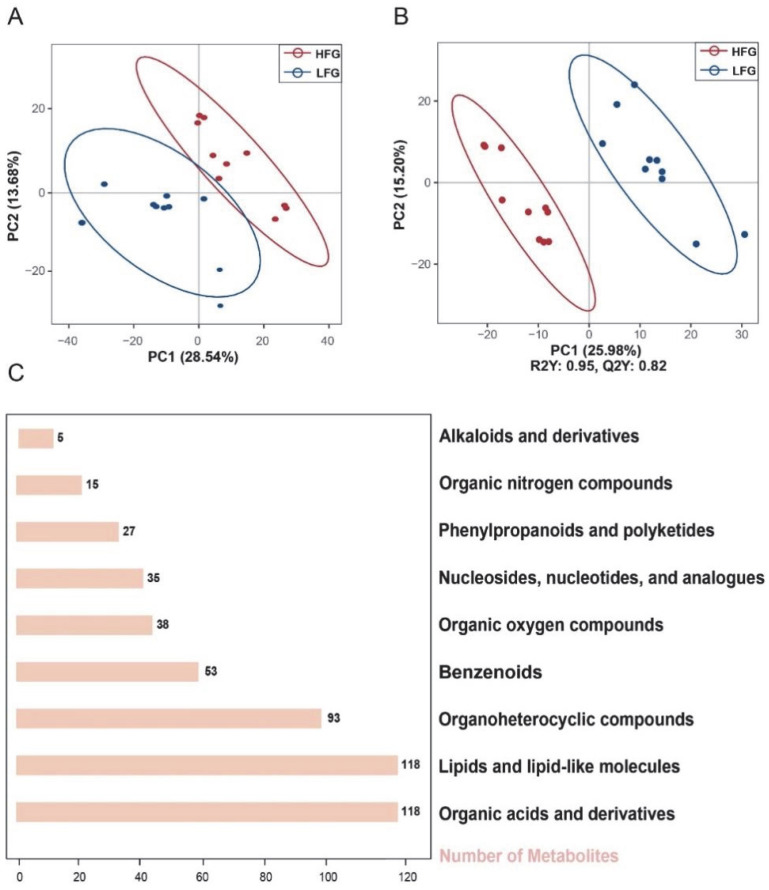
Classification of Metabolites in Semen Samples from HFG and LFG. (**A**) Principal component analysis (PCA). (**B**) Orthogonal partial least squares discriminant analysis (OPLS-DA). Red dots represent samples from the HFG, while blue dots represent samples from the LFG. (**C**) Chemical classification of metabolites and the number of metabolites within each category.

LC-MS analysis identified a total of 960 metabolites in the semen samples. PCA and OPLS-DA clearly separated the HFG and LFG (Figure 1A,B), demonstrating significant compositional differences. Metabolites were annotated using HMDB and categorized into nine chemical classes, Alkaloids and derivatives (0.99%), Organic nitrogen compounds (2.99%), Phenylpropanoids and polyketides (5.38%), Nucleosides, nucleotides, and analogs (6.97%), Organic oxygen compounds (7.56%), Benzenoids (10.56%), Organoheterocyclic compounds (18.53%), Lipids and lipid-like molecules (23.51%), and Organic acids and derivatives (23.51%) (Figure 1C).

### 3.3. Screening of Differential Metabolites Between HFG and LFG

**Figure 2:**
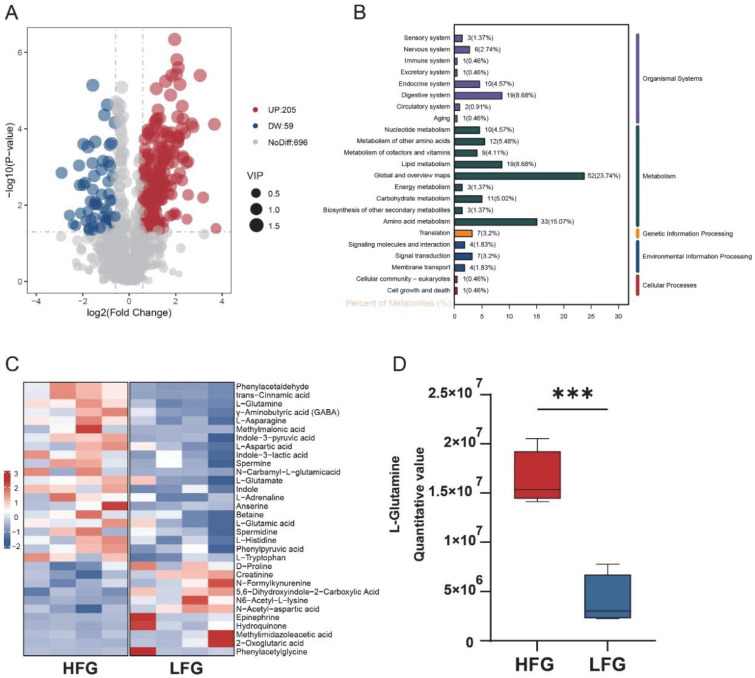
Differential Metabolites in Semen Samples from HFG and LFG. (**A**) Volcano plot of differential metabolites. Red circles represent upregulated metabolites, blue circles represent downregulated metabolites, and gray circles represent metabolites with no significant difference between the groups. The x-axis shows the log_2_ fold change (FC) values, with dashed lines at log_2_(1.5) and log_2_(0.667). The y-axis shows the negative logarithm of the P-values to the base 10, with a dashed line at −lg (0.05). The size of each circle indicates its VIP value. (**B**) Distribution of differential metabolites in KEGG metabolic pathways. Cyan represents Organismal Systems, green represents Metabolism, yellow represents Genetic Information Processing, blue represents Environmental Information Processing, and red represents Cellular Processes. (**C**) Abundance of differential metabolites in the amino acid pathway. Each row represents a different metabolite, and each column represents a sample. Red indicates upregulated metabolite abundance relative to the average, while blue indicates downregulated abundance. (**D**) Levels of L-Gln in both groups. Red represents HFG, and blue represents LFG. The line inside each box represents the median, the box boundaries represent the 25th percentile (Q1) and the 75th percentile (Q3), and the whiskers extend to 1.5 times the interquartile range. *** indicates a *p* value < 0.001.

Using the LFG as the control, 264 differential metabolites were identified in the HFG, with 205 upregulated and 59 downregulated compounds (Figure 2A). KEGG pathway annotation revealed that lipid metabolism (6.88%) and amino acid metabolism (15.07%) were prominently represented (Figure 2B). Notably, metabolites such as L-Gln, L-glutamate, γ-aminobutyric acid (GABA), and L-asparagine were significantly upregulated in the HFG, with L-Gln showing a particularly high abundance compared to the LFG (Figure 2C,D). In contrast, metabolites including 2-oxoglutaric acid and hydroquinone were more abundant in the LFG. These findings underscore the potential role of L-Gln in enhancing cryotolerance.

### 3.4. Effect of L-Glutamine on Sperm Motility in Frozen–Thawed Sperm

**Figure 3:**
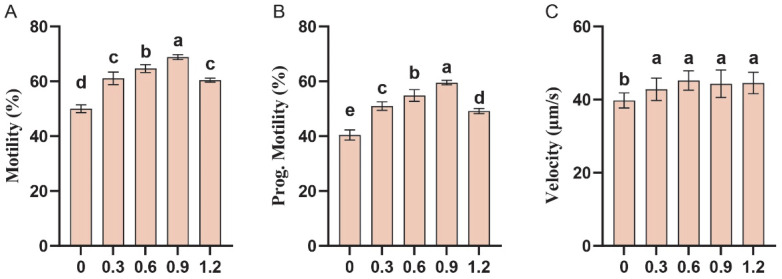
Impact of Different Concentrations of L-Gln on the Motility of Post-Thaw Sperm. (**A**) Sperm motility. (**B**) Sperm progressive motility. (**C**) Sperm velocity. The x-axis represents the final concentrations of L-Gln in the system. The bar graphs show the mean ± standard deviation (mean ± SD). Identical letters indicate no significant difference (*p* > 0.05), while different letters between bars indicate significant differences (*p* < 0.05).

To validate the role of L-Gln as an endogenous cryoprotectant, L-Gln was added to fresh semen before dilution, equilibration, and freezing. Post-thaw assessments revealed that increasing L-Gln concentrations significantly improved both overall sperm motility and progressive motility, with the peak effect observed at 0.9 mg/mL, where motility increased by 18.83% and progressive motility by 19.09% compared to the control group (*p* < 0.05; Figure 3A,B). Although no significant differences were observed between treatment groups, sperm velocity was significantly higher than that of the control group and exhibited a slight increasing trend with higher concentrations of L-Gln (*p* < 0.05; Figure 3C).

### 3.5. Effect of L-Glutamine on Antioxidant Capacity in Frozen–Thawed Sperm

**Figure 4:**
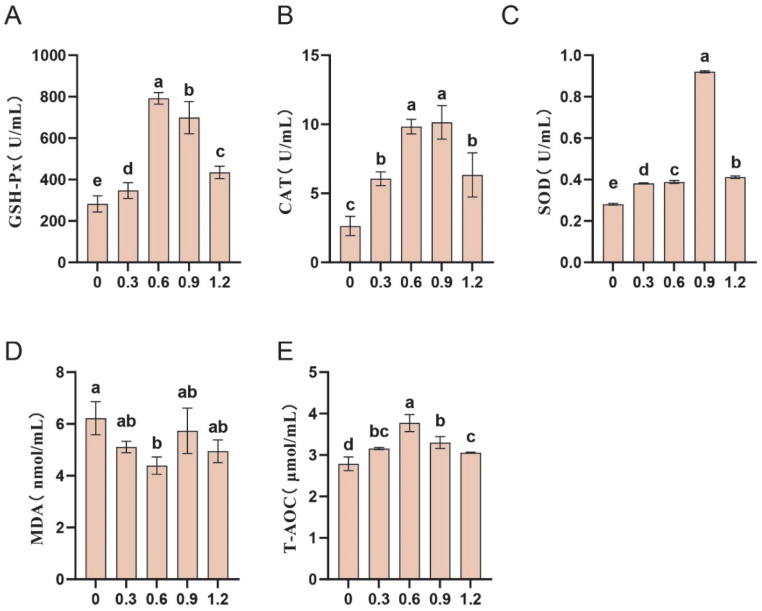
Effect of Different Concentrations of L-Gln on the Antioxidant Capacity of Post-Thaw Sperm. (**A**) Sperm GSH-Px content. (**B**) Sperm CAT content. (**C**) Sperm SOD content. (**D**) Sperm MDA content. (**E**) Total antioxidant capacity of Sperm. The x-axis represents the final concentrations of L-Gln in the system. The bar graphs show the mean ± standard deviation (mean ± SD). Identical letters indicate no significant difference (*p* > 0.05), while different letters between bars indicate significant differences (*p* < 0.05).

We further assessed the effects of L-Gln on antioxidant capacity. The results indicated that the addition of L-Gln significantly increased the activities of CAT, SOD, and GSH-Px (*p* < 0.05; Figure 4A–C), while reducing MDA content (*p* < 0.05; Figure 4D). The highest antioxidant capacity was observed in sperm at an L-Gln concentration of 0.6 mg/mL, where T-AOC reached its peak (*p* < 0.05; Figure 4E).

### 3.6. Effect of L-Glutamine on Membrane Integrity in Frozen–Thawed Sperm

**Figure 5:**
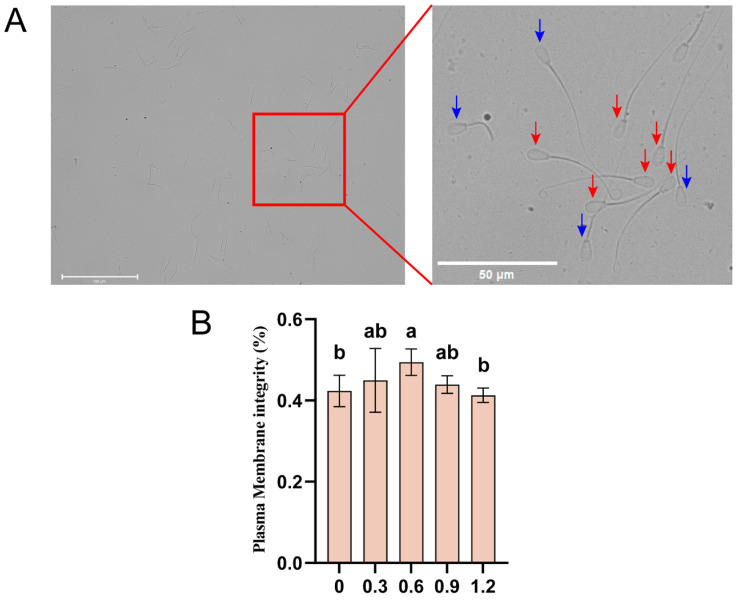
Effect of Different Concentrations of L-Gln on the Plasma Membrane Status of Post-Thaw Sperm. (**A**) Sperm plasma membrane status. Blue arrows indicate incomplete membrane structures, while red arrows indicate intact membrane structures. (**B**) Sperm plasma membrane integrity. The x-axis represents the final concentrations of L-Gln in the system. The bar graphs show the mean ± standard deviation (mean ± SD). Identical letters indicate no significant difference (*p* > 0.05), while different letters between bars indicate significant differences (*p* < 0.05).

To further assess the protective effects of L-Gln on sperm structure, we evaluated the integrity of the plasma membrane in post-thaw sperm (Figure 5A). L-Gln significantly improved sperm plasma membrane integrity, particularly at a concentration of 0.6 mg/mL, where membrane integrity was notably enhanced (*p* < 0.05; Figure 5B).

### 3.7. Effect of L-Glutamine on Acrosomal Integrity in Frozen–Thawed Sperm

**Figure 6:**
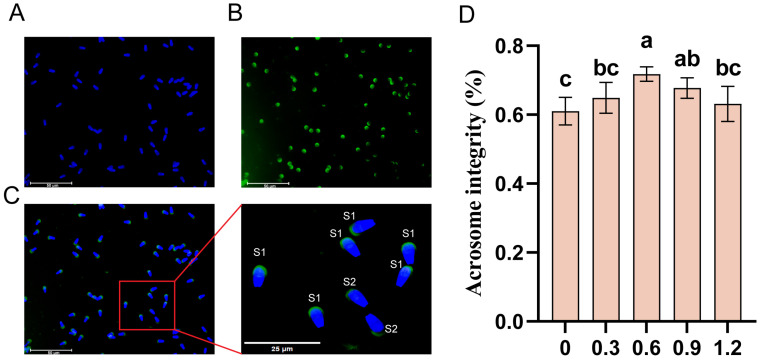
Effect of Different Concentrations of L-Gln on Acrosome Status of Post-Thaw Sperm. (**A**) DAPI Staining (**B**) FITC-PNA Staining (**C**) Merge, where “S1” indicates sperm with an integral acrosome; “S2” indicates sperm with a damaged acrosome. (**D**) Acrosome Integrity Rate. The x-axis represents the final concentrations of L-Gln in the system. The bar graphs show the mean ± standard deviation (mean ± SD). Identical letters indicate no significant difference (*p* > 0.05), while different letters between bars signify significant differences (*p* < 0.05).

FITC-PNA staining was performed to quantify the proportion of sperm with intact acrosomes and calculate the acrosome integrity rate (Figure 6A–C). Compared to the control group, L-Gln at concentrations of 0.6 mg/mL and 0.9 mg/mL significantly protected acrosome integrity (*p* < 0.05), with the most pronounced effect observed at 0.6 mg/mL (Figure 6D).

### 3.8. Effect of L-Glutamine on Energy Metabolism and AMPK Phosphorylation in Frozen–Thawed Sperm

**Figure 7:**
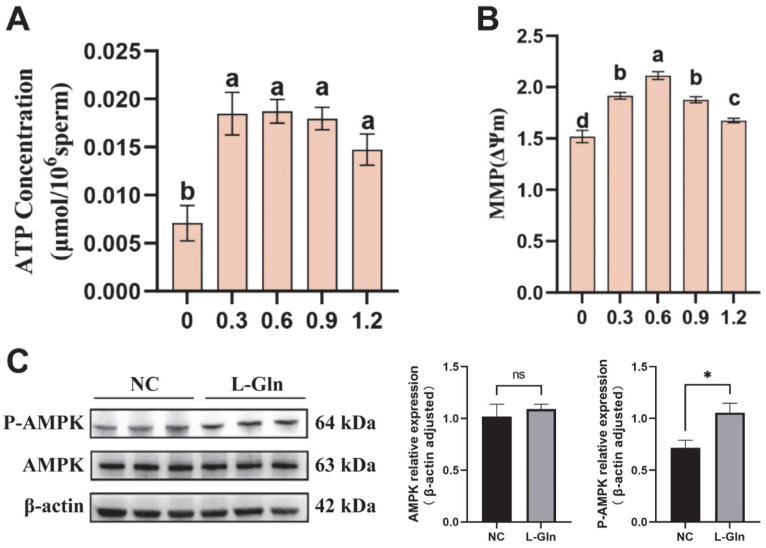
Effect of Different Concentrations of L-Gln on Energy Metabolism in Post-Thaw sperm. (**A**) sperm ATP content. (**B**) Mitochondrial membrane potential of sperm. The x-axis represents the final concentrations of L-Gln. The bar graphs show the mean ± standard deviation (mean ± SD). Identical letters indicate no significant differences (*p* > 0.05), while different letters between bars signify significant differences (*p* < 0.05). (**C**) Expression levels of AMPK, p-AMPK, and β-actin in sperm from the control group and the 0.6 mg/mL treatment group. “ns” denotes no significant difference, and “*” indicates a significant difference (*p* < 0.05).

This study evaluated the effect of L-Gln on sperm ATP content and mitochondrial membrane potential after semen cryopreservation. The results showed that L-Gln significantly increased post-thaw sperm ATP content compared to the control group (*p* < 0.05), although no significant differences were observed between treatment groups (*p* > 0.05) (Figure 7A). L-Gln also significantly enhanced post-thaw sperm mitochondrial membrane potential (*p* < 0.05), with the maximal effect observed at a concentration of 0.6 mg/mL (Figure 7B). Western blot analysis showed that while total AMPK levels remained unchanged (*p* > 0.05), phosphorylation of AMPK (p-AMPK) was significantly upregulated in the 0.6 mg/mL group Figure 7C).

## 4. Discussion

Semen consists of sperm and seminal plasma, where small-molecule metabolites play crucial roles in sperm motility, energy acquisition, acrosome reaction, and fertilization [21]. However, the content of small-molecule metabolites varies significantly among different semen samples, which may lead to differences in the semen cryopreservation effect [22]. This study evaluated the motility of fresh sperm from eight healthy Qinchuan bulls before and after freezing, finding that freeze–thaw resulted in significantly less impairment of sperm motility in HFG compared to LFG. Further analysis of the semen metabolome revealed that lipids and lipid-like molecules (23.51%), as well as organic acids and derivatives (23.51%), were the predominant metabolites in the semen of both groups. Lipid molecules are closely associated with membrane fluidity and integrity, which are essential for maintaining sperm motility during cryopreservation [23]. Organic acids are likely involved in energy metabolism and contribute to antioxidant capacity. Amino acids, as key components of organic acids, play unique roles in cryotolerance [24]. Studies have shown that specific amino acids can enhance sperm survival by regulating osmotic balance, reducing ice crystal formation, and alleviating oxidative stress. For example, aspartic acid protects sperm from oxidative stress by reducing lipid peroxidation and DNA fragmentation, thereby improving embryonic development potential [25]. Histidine helps maintain intracellular pH stability, enhance antioxidant enzyme activity, and promote carnosine synthesis, thereby alleviating oxidative stress [26]. Tryptophan and its metabolites, through conversion into the potent antioxidant melatonin, scavenge ROS, improve sperm quality, and enhance in vivo fertilization rates [27].

The combined contribution of Amino acid metabolism (33 metabolites, 15.07%) and Amino acid-related pathways (12 metabolites, 4.57%) suggests that amino acids play a crucial role in the metabolic network of cryopreserved semen. This supports the previously discussed roles of amino acids in osmoregulation, antioxidation, and energy metabolism. Further analysis of differential metabolites in amino acid metabolic pathways revealed that 22 metabolites, including L-glutamine and L-asparagine, were significantly upregulated in HFG, while 11 metabolites such as 2-ketoglutarate and hydroquinone showed significant downregulation. Notably, L-Gln has been demonstrated to promote the production of GSH and increase the activity of GSH-Px in rabbit sperm, protecting sperm from oxidative stress damage [28]. Additionally, L-Gln not only directly participates in the TCA cycle but also undergoes decarboxylation to produce GABA, which is further converted into succinate to enter the TCA cycle, thus providing sustained energy support ADDIN [29]. Similarly, L-asparagine is metabolized into aspartate, an important substrate for the TCA cycle, contributing to energy metabolism. These amino acids may be interconnected through the TCA cycle, acting synergistically to enhance the cryotolerance of semen in the HFG [30]. However, 2-Oxoglutaric acid, a critical intermediate in the TCA cycle, is generated through the oxidation of isocitrate and can also be derived from glutamine metabolism. It serves as a central molecule in amino acid metabolism, facilitating the interconversion between glutamate and glutamine, thus linking the TCA cycle and amino acid metabolism [31]. The accumulation of 2-Oxoglutaric acid in the LFG may result from reduced TCA cycle efficiency or impaired glutamine and glutamate metabolism. Hydroquinone, on the other hand, may be a byproduct of oxidative stress generated by ROS during intracellular metabolism. Its relative enrichment in the LFG could indicate insufficient antioxidant capacity in LFG sperm. This deficiency may stem from reduced coupling efficiency between amino acid metabolism and the TCA cycle, limiting the cell’s ability to generate sufficient antioxidant defenses and compromising sperm cryotolerance. Given the excellent performance of L-Gln in antioxidant stress and energy metabolism, subsequent experiments systematically investigated its effects on sperm cryotolerance in terms of motility, acrosome and plasma membrane integrity, antioxidant capacity, and energy metabolism.

The plasma membrane and acrosome are important structures of sperm. The sperm plasma membrane serves as both a physical barrier and a critical functional structure for sperm motility, fertilization, signal transduction, and antioxidative protection. Additionally, the acrosome is vital for sperm-oocyte binding [32,33]. Freezing-induced ice crystal formation and oxidative stress can compromise membrane structure [34]. Furthermore, excessive ROS under oxidative stress can attack unsaturated fatty acids in the sperm plasma membrane, leading to lipid peroxidation and generation of products like MDA, which disrupts membrane integrity and fluidity, and subsequently impairs sperm motility and fertilization potential [35]. In this study, L-Gln significantly reduced the content of MDA, indicating its effective alleviation of lipid peroxidation and highlighting its protective role in sperm membrane integrity.

Enhancing antioxidant capacity is a key mechanism for sperm to resist oxidative stress. As a precursor of GSH, L-Gln promotes GSH synthesis, scavenges ROS generated during cryopreservation, and protects sperm structure and function ADDIN [18]. On the other hand, L-Gln activates the Nrf2 signaling pathway to enhance the expression of antioxidant enzymes [17]. In this study, L-Gln effectively alleviated sperm oxidative damage by enhancing the activities of antioxidant enzymes such as SOD, GSH-Px, and CAT, reducing intracellular ROS levels, and mitigating lipid peroxidation.

Energy metabolism is crucial for sperm function. Mitochondria serves as the cellular powerhouses vital for sperm survival and function during cryopreservation [36]. During freezing, mitochondrial membranes are prone to damage, resulting in reduced membrane potential and impaired ATP production [37,38]. AMPK acts as an upstream target for mitochondrial quality control, modulating mitochondrial fusion and fission to maintain mitochondrial dynamic balance [39]. Additionally, AMPK serves as a cellular energy sensor. Activated by changes in the ATP/AMP ratio, it regulates cellular ATP levels and energy homeostasis by inhibiting anabolism and promoting catabolic processes such as glycolysis, fatty acid β-oxidation, and autophagy [40]. Furthermore, studies have found that AMPK can modulate the expression of multiple antioxidant enzymes via the AMPK/Nrf2 signaling pathway, thereby enhancing sperm’s ability to repair oxidative damage [41]. The increase in AMPK phosphorylation level, mitochondrial membrane potential, and antioxidant enzyme activities indicates that L-Gln can stabilize mitochondrial structure and enhance antioxidant enzyme activities by activating the AMPK signaling pathway, thereby improving post-thaw sperm energy homeostasis and antioxidant defense system.

Beyond its role as a cryoprotectant when added before freezing, L-Gln may also provide post-thaw benefits. After thawing, sperm cells experience increased oxidative stress, membrane destabilization, and mitochondrial dysfunction. Supplementing L-Gln post-thaw may replenish intracellular substrates for glutathione synthesis, enhance antioxidant defenses, and mitigate ROS induced damage.

## 5. Conclusions

This study systematically elucidates the differential metabolite profiles between semen with high and low freezability from Qinchuan cattle, and identifies L-Gln as a potential endogenous cryoprotectant through metabolomic analysis. Further experimental validation demonstrated that the addition of L-Gln to the freezing diluent significantly enhanced post-thaw sperm motility, antioxidant capacity, membrane structural integrity, and energy metabolism. These effects are likely mediated by L-Gln’s role as a precursor for glutathione synthesis and a key substrate in the TCA cycle, which together boost antioxidant system activity and energy metabolism. The optimal concentration of L-Gln was found to be 0.9 mg/mL, resulting in a 19.09% increase in progressive motility. These findings suggest the potential application of L-Gln in improving semen freezing efficiency, which provides valuable insights and a theoretical foundation for the development of endogenous cryoprotectants in cattle.

## Figures and Tables

**Table 1:** The Sperm Motility Parameters of the LFG and HFG.

Group	Fresh Sperm Motility (%)	Fresh Sperm PR.MOT (%)	Frozen–Thawed Sperm Motility (%)	Frozen–Thawed Sperm PR.MOT (%)
LFG	81.64 ± 4.10 ^a^	71.6 ± 3.33 ^a^	25.11 ± 1.29 ^a^	16.1 ± 1.45 ^a^
HFG	79.91 ± 4.07 ^a^	76.6 ± 3.33 ^a^	56.57 ± 1.29 ^b^	47.3 ± 1.45 ^b^

Values with same lowercase superscript in the same column mean no significant difference (*p* > 0.05), while the values with different lowercase superscript mean significant differences (*p* < 0.05). Abbreviation: PR.MOT, progressive motility.

## Data Availability

The raw data supporting the conclusions of this article will be made available by the authors on request.

## Abbreviations

The following abbreviations are used in this manuscript:

L-Gln	L-glutamine
HFG	high-freezing group
LFG	low-freezing group
ROS	reactive oxygen species
LC-MS	liquid chromatography–mass spectrometry
PR.MOT	Progressive motility
MDA	Malondialdehyde
CAT	catalase
GSH-Px	glutathione peroxidase
SOD	superoxide dismutase
T-AOC	total antioxidant capacity
MMP	Mitochondrial Membrane Potential
ATP	Adenosine triphosphate
AMPK	Adenosine 5′-monophosphate-activated protein kinase
Nrf2	Nuclear factor e2-related factor 2
TCA	Tricarboxylic acid
GABA	γ-aminobutyric acid
